# Editorial: Microbial interactions and survival mechanisms in chronic respiratory infections

**DOI:** 10.3389/fmicb.2024.1387518

**Published:** 2024-02-29

**Authors:** Que Chi Truong-Bolduc, Chun-Hsing Liao, Hidemasa Nakaminami, Jacob Strahilevitz, Leon G. Leanse

**Affiliations:** ^1^Massachusetts General Hospital, Harvard Medical School, Boston, MA, United States; ^2^School of Medicine, College of Medicine, National Yang Ming Chiao Tung University, Taipei City, Taiwan; ^3^Department of Microbiology, School of Pharmacy, Tokyo University of Pharmacy and Life Sciences, Hachioji, Japan; ^4^Department of Clinical Microbiology and Infectious Diseases, Department of Medicine, Hadassah-Hebrew University Medical Center, Jerusalem, Israel; ^5^Health and Sport Sciences Hub, University of Gibraltar, Europa Point, Gibraltar; ^6^Wellman Center for Photomedicine, Massachusetts General Hospital, Harvard Medical School, Boston, MA, United States

**Keywords:** microbiome, interaction, survival, CRDs, bacteria, infections, antibiotics

Chronic respiratory diseases (CRDs) such as cystic fibrosis (CF), non CF-bronchiectasis and asthma are common non-communicable diseases that affect the airways and other lung structures (Labaki and Han, 2020), and infections in CRD patients are caused by various pathogens (Cullen and McClean, 2015). Because of structural and functional dysfunctions of the lungs, patients are unable to clear the pathogens from the respiratory tract, thereby leading to the development of chronic phase of infections which are more difficult to treat by antibiotics. CRD patients might receive prolonged and repeated antibiotics, which hasten the evolution of bacteria.

Within a chronic infection site, the microbial community exercises various modalities of inter-species interactions resulting in a balance between competition and cooperation which ultimately leads to negative outcome for the patients (Limoli et al., 2016).

Cystic fibrosis (CF) is an autosomal recessively inherited disorder that affects major organs including the lungs. CF patients usually exhibit a thick mucus layer coating the airways that constitutes the habitat of many microbes including the two most common pathogens *S. aureus* and *P. aeruginosa*. Whereas early invading *P. aeruginosa* is known to secrete compounds that inhibit the growth of *S. aureus*, late *P. aeruginosa* strains, which exhibit less virulence, establish a coexistence with *S. aureus* (Pallett et al., 2019).

Camus et al. provide a review article on the subject of *P. aeruginosa* and *S. aureus* co-infection. They indicated that gene alterations and reduction of virulence factors via adjustment of specific regulators are some pathoadaptive changes that enhance the survival and persistence of *P. aeruginosa* at the infection site. These adaptive modifications lead to the emergence of more resistant to antibiotics but less virulent *P. aeruginosa* that show a reduced anti-staphylococcal activity, promoting a lifestyle of coexistence with *S. aureus*. Late *S. aureus* are frequently auxotrophic and survive as small colony variant (SCV) that are resistant to antibiotics and associated with worse respiratory complications in CF patients. The authors emphasize that *S. aureus* also influence the adaptation of *P. aeruginosa* to the CF environment and highlight the research gap in the long-term co-evolution of *S. aureus* and *P. aeruginosa* in CF patients and its impact on better infection management.

Various opportunistic microorganisms also colonize and infect the airways of CF patients, contributing to their morbidity and mortality (Cuthbertson et al., 2020). The frequent use of antibiotics affects both the targeted pathogens as well as the surrounding microbiota leading to alterations of the microbial community dynamics. CF patients are also treated with CFTR (CF transmembrane conductance regulator) modulators that adequately improve the pathophysiology and the CF environment to promote a healthy microbiota (Dawood et al., 2022; Miller et al., 2022; Sosinski et al., 2022). Understanding how multiple factors affect the microbiota of the CF airways is crucial in the attempt to manage the disease. Ho et al. developed a Lotka-Volterra model to analyze the ecological interactions between microbial taxa recorded in the UK CF registry during a period of 12 years (2008–2020). The authors report that certain medications appear to influence the “gut-lung axis” as well as the bacterial biofilm formation within the airway mucus which possibly impact the ecology of the CF airway. Furthermore, the authors suggest that CFTR modulators may also influence the gut physiology.

Non-CF bronchiectasis is a condition characterized by abnormal widened and inflamed lung airways that is associated with chronic cough, mucus production, and recurrent infections (McShane et al., 2013). The most common pathogen causing airway inflammation, exacerbation, leading to recurrent hospitalizations in non-CF bronchiectasis is *P. aeruginosa*, but other microbes, such as non-tuberculous mycobacteria (NTM) also play a role in these infections (Richardson et al., 2019). The implication of NTM is still unclear despite recent study indicating an association between NTM infection and patient body weight loss (Faverio et al., 2016). Lin et al. report on the impacts of NTM in non-CF bronchiectasis using a 16-year cohort study in Taiwan. The authors demonstrated that NTM in combination with *P. aeruginosa* or with fungi caused more exacerbations and respiratory failure in non-CF bronchiectasis patients. This is an important finding which requires further investigations.

Asthma is a common CRD that affects all age groups and is marked by inflammation of the bronchial tubes with sticky secretions insides the tubes. The pathogenesis of asthma is complex. Asthma is a chronic disease with significant economic impact due to long-term treatment (Kuruvilla et al., 2019). The comprehensive review by Galeana-Cadena et al. describes the roles of the microbiome in relation to the phenotypes and endotypes (mechanistic pathway leading to asthma) of asthma throughout human growth and development. The authors emphasize that the microbiome influences early-life immune development and modulation. They also point out the limitations of the studies conducted thus far which address these issues, due to difficulty obtaining relevant samples from certain areas of the human body. The authors express the needs to include virome and mycobiome in future studies investigating the mechanisms underlying these interactions.

Communications between host and microbiota involve components of the host immune system and bacterial antigens and exoproducts (Albillos et al., 2020). Xiao et al. provide a comprehensive review on the significance of bacterial membrane vesicles (MVs) as a communication vehicle between host and bacteria. The authors offer an analysis of the role of various classes of MVs in transport and immune response activation and raise the possibility of using MVs as a therapeutic approach, with applications in vaccines, in making adjuvant, or in designing a drug carrier.

Lastly, Yang et al. provide the evidence of a relationship between the quorum-sensing system and antibiotic resistance of *P. aeruginosa*. The authors demonstrate that clinical *P. aeruginosa* display phenotypic heterogeneity, and that *P. aeruginosa* isolates exhibiting broader antibiotic resistance also display a reduction in LasR-dependent extracellular proteases. These findings offer future strategies to address *P. aeruginosa* antibiotic resistance in CRDs.

In conclusion, the reviews and articles published in this Research Topic shed additional light on interactions between microbes infecting patients with CRDs and the bacterial mechanisms of adaptation to persist at the infection sites. The authors also provide insights into future development of therapeutic strategies and the importance of microbiome, mycobiome, and virome on human wellbeing.

## Author contributions

QT-B: Conceptualization, Formal analysis, Writing – original draft, Writing – review & editing. C-HL: Writing – review & editing. HN: Writing – review & editing. JS: Writing – review & editing. LL: Writing – review & editing.

## References

[B1] Albillos A. Gottardi A. Rescigno M. (2020). The gut-liver axis in liver disease: Pathophysiological basis for therapy. J. Hepatol. 72, 558–577. 10.1016/j.jhep.2019.10.003 31622696

[B2] Cullen L. McClean S. (2015). Bacterial adaptation during chronic respiratory infections. Pathogens 4, 66–89. 10.3390/pathogens4010066 25738646 PMC4384073

[B3] Cuthbertson L. Walker A. Oliver A. Roger G. Rivett D. Hampton T.. (2020). Lung function and microbiota diversity in cystic fibrosis. Microbiome 8, 45. 10.1186/s40168-020-00810-3 32238195 PMC7114784

[B4] Dawood S. N. Rabih A. M. Niaj A. Raman A. Uprety M. Calero M. J.. (2022). Newly discovered cutting-edge triple combination cystic fibrosis therapy: a systematic review. Cureus 14, e29359. 10.7759/cureus.29359 36284811 PMC9583755

[B5] Faverio P. Stainer A. Bonati G. Zucchetti S. Simonetta E. Lapadula G.. (2016). Characterizing Non-Tuberculous Mycobacteria Infection in Bronchiectasis. Int. J. Mol. Sci. 17 10.3390/ijms17111913 27854334 PMC5133910

[B6] Kuruvilla M. E. Lee F. E. Lee G. B. (2019). Understanding asthma phenotypes, endotypes, and mechanisms of disease. Clin. Rev. Allergy Immunol. 56, 219–233. 10.1007/s12016-018-8712-1 30206782 PMC6411459

[B7] Labaki W. W. Han M. K. (2020). Chronic respiratory diseases: a global view. Lancet Respir. Med. 8, 531–533. 10.1016/S2213-2600(20)30157-0 32526184 PMC8034823

[B8] Limoli D. H. Yang J. Khansaheb M. K. Helfman B. Peng L. Stecenko A. A.. (2016). *Staphylococcus aureus* and *Pseudomonas aeruginosa* co-infection is associated with cystic fibrosis-related diabetes and poor clinical outcomes. Eur. J. Clin. Microbiol. Infect. Dis. 35, 947–953. 10.1007/s10096-016-2621-0 26993289

[B9] McShane P. J. Naureckas T. Tino G. Strek M. (2013). Non-cystic fibrosis bronchiectasis. Am. J. Respir. Crit. Care Med. 188, 647–656. 10.1164/rccm.201303-0411CI 23898922

[B10] Miller A. C. Harris L. M. Cavanaugh J. E. Alaiwa M. A. Stoltz D. A. Hornick D. B.. (2022). The rapid reduction of infection-related visits and antibiotic use among people with cystic fibrosis after starting elexacaftor-tezacaftor-ivacaftor. Clin. Infect. Dis. 75, 1115–1122. 10.1093/cid/ciac117 35142340 PMC9525072

[B11] Pallett R. Leslie L. J. Lambert P. A. Milic I. Devitt A. Marshall L. J. (2019). Anaerobiosis influences virulence properties of *Pseudomonas aeruginosa* cystic fibrosis isolates and the interaction with *Staphylococcus aureus*. Sci. Rep. 9, 6748. 10.1038/s41598-019-42952-x 31043640 PMC6494883

[B12] Richardson H. Dicker A. Barcley H. Chalmers J. D. (2019). The microbiome in bronchiectasis. Eur. Respir. Rev. 28, 190048. 10.1183/16000617.0048-2019 31484665 PMC9489022

[B13] Sosinski L. M. Martin H. Neugebauer K. A. Ghuneim L-A. J. Guzior D. V. Castillo-Bahena A.. (2022). A restructuring of microbiome niche space is associated with Elexacaftor-Tezacaftor-Ivacaftor therapy in the cystic fibrosis lung. J. Cyst. Fibros. 21, 996–1005. 10.1016/j.jcf.2021.11.003 34824018 PMC9124239

